# The T Allele of the HNMT C314T Polymorphism Is Associated with a Reduced Risk of Idiopathic Parkinson’s Disease in Mexican Patients

**DOI:** 10.3390/biomedicines14040861

**Published:** 2026-04-09

**Authors:** Antonio Bueno-Nava, Diana-Karina Díaz-Hernández, Rogelio Paniagua-Pérez, Paul Carrillo-Mora, José-Antonio Martínez-Cortez, Claudia Hernández-Arenas, Saúl-Renán León-Hernández, Adriana Olmos-Hernández, Antonio Verduzco-Mendoza, Alberto Avila-Luna, Arturo Gálvez-Rosas

**Affiliations:** 1 División de Neurociencias, Instituto Nacional de Rehabilitación Luis Guillermo Ibarra Ibarra, Mexico City 14389, Mexicopcarrillo@inr.gov (P.C.-M.);; 2 Servicio de Bioquímica, Instituto Nacional de Rehabilitación Luis Guillermo Ibarra Ibarra, Mexico City 14389, Mexico; 3 Servicio de Neurología, Instituto Nacional de Rehabilitación Luis Guillermo Ibarra Ibarra, Mexico City 14389, Mexico; 4 Servicio de Daño Cerebral Aquirido, Instituto Nacional de Rehabilitación Luis Guillermo Ibarra Ibarra, Mexico City 14389, Mexico; 5 Unidad de Apoyo a la Investigación, Instituto Nacional de Rehabilitación Luis Guillermo Ibarra Ibarra, Mexico City 14389, Mexico; 6 Servicio de Bioterio y Cirugía Experimental, Instituto Nacional de Rehabilitación Luis Guillermo Ibarra Ibarra, Mexico City 14389, Mexico; adrianaolmos05@yahoo.com.mx (A.O.-H.);

**Keywords:** Parkinson’s disease, histamine N-methyltransferase gene, restriction fragment length polymorphism

## Abstract

**Introduction:** The histaminergic pathway has been implicated in Parkinson’s disease (PD). Histamine is metabolized by histamine N-methyltransferase (HNMT), and the gene encoding this enzyme has a C314T polymorphism, in which cytosine is replaced by thymine. This results in reduced enzymatic activity. **Objective:** To analyze the C314T polymorphism of the HNMT gene in Mexican patients with idiopathic PD. **Materials and Methods:** In this study, peripheral blood samples were collected from patients with PD and healthy controls for genomic DNA extraction. HNMT genotyping was performed using the restriction fragment length polymorphism (RFLP) technique. Quantitative variables were compared using Student’s *t* test, and categorical variables were compared using Pearson’s χ^2^ test. The risk of PD was estimated using odds ratios (ORs) and 95% confidence intervals (CIs). **Results:** According to the results of the bivariate analysis, compared with the controls, the patients were significantly older (*p* = 0.001) and had a higher incidence of hypertension (*p* = 0.020). HNMT RFLP analysis suggested an association between the C allele and PD development, with an OR (95% CI) of 7.424 (0.866–63.646). In contrast, the T allele appeared to confer a protective effect, with an OR of 0.134. In the age-adjusted Mantel—Haenszel stratified analysis of the HNMT C314T polymorphism, the C allele was identified as a risk factor for PD development in this small cohort, with an OR (95% CI) of 12.0 (0.8–160.4; *p* = 0.041). **Conclusions:** Advanced age, hypertension, and the C allele of the HNMT gene were associated with an increased risk of PD, whereas the T allele appeared to be associated with a protective role.

## 1. Introduction

Parkinson’s disease (PD) is a chronic, progressive neurodegenerative disorder in which a neurological diagnosis typically occurs only after the onset of motor symptoms, including resting tremors, bradykinesia, muscle rigidity, and postural instability [1,2]. By this stage, more than 70% of the dopaminergic neurons in the substantia nigra pars compacta (SNc) have already degenerated, and the pathological hallmark of PD is the presence of Lewy bodies [3,4]. The etiology of PD remains largely unknown, although epidemiological evidence suggests contributions from both genetic and environmental factors [5,6,7]. However, regardless of the underlying etiology, several key molecular pathophysiological events have been described, including α-synuclein aggregation, mitochondrial dysfunction, abnormalities in the ubiquitin–proteasome and autophagy–lysosomal systems, neuroinflammation, and more recently, glycemic variability [3,8,9].

The histaminergic system has also been implicated in PD. In the periphery, histamine is released by mast cells, whereas in the central nervous system (CNS), it is released by histaminergic neurons of the tuberomammillary nucleus, which is located in the posterior basal hypothalamus. Within the brain, histaminergic projections extend to multiple regions, including the thalamus, hippocampus, striatum, amygdala, and cerebral cortex [10,11,12]. Histamine, a biogenic amine, is involved in neurodegeneration and neurotoxicity [13,14]. It is synthesized from the amino acid histidine by L-histidine decarboxylase and can cause selective damage to the dopaminergic neurons of the SNc through inflammatory mechanisms [13,14]. Moreover, alterations in the morphology and increased density of histaminergic fibers in the SNc have been observed in PD patients [11,15]. Histamine is metabolized via two main pathways: (a) oxidation by diamine oxidase (DAO) and (b) methylation by histamine N-methyltransferase (HNMT). Elevated histamine levels have been detected in the blood of patients with PD [16,17], along with increased concentrations of histamine metabolites in the cerebrospinal fluid [18,19]. Brains of patients with PD exhibit an increase in the density of histaminergic fibers in the degenerated areas of the substantia nigra as a compensatory response [15]. Moreover, elevated HNMT mRNA levels have been described in both the substantia nigra and putamen [20]. Collectively, these findings suggest that alterations in the histaminergic system may be associated with PD.

As part of the efforts to identify additional factors involved in PD, a single-nucleotide polymorphism (SNP) has been reported in the HNMT gene [21,22]. The resulting gene products may play a functional role in PD, as the encoded enzyme participates in histamine metabolism in the human brain, potentially influencing local histamine levels in the CNS [23].

Given that the HNMT SNP rs11558538 (C314T) in exon 4 has not been investigated in the Mexican population, the aim of the present study was to analyze this variant in Mexican patients with idiopathic PD.

## 2. Materials and Methods

All participants were Mexican mestizos with at least three generations of ancestry from the same place of origin and provided written informed consent prior to participation in the study. We evaluated a cohort of 18 patients with idiopathic PD who were recruited from the Neurology Department at the Instituto Nacional de Rehabilitación Luis Guillermo Ibarra Ibarra (INR-LGII). Patients met the established clinical diagnostic criteria and exhibited motor symptoms consistent with PD, in accordance with the recommendations of the Neurology and Acquired Brain Injury Services. Information on comorbid conditions was obtained from a structured questionnaire in which participants reported the presence or absence of concomitant diseases (Table 1). Some patients had been previously diagnosed at other institutions, whereas others were long-term follow-up patients at the INR-LGII. The motor symptoms assessed included bradykinesia, rigidity, tremor, and postural instability. Functional status was evaluated using the Hoehn and Yahr rating scale [23,24].

Healthy controls (*n* = 20) were recruited from the INR-LGII outpatient clinic. Control subjects were originally planned to be randomly selected from patients’ spouses and hospital staff; however, this was not feasible because patients were widowed and the relevant hospital staff had already retired. Therefore, healthy controls were ultimately recruited from the general population. A medical examination was performed to confirm that the controls were in good health. The study protocol was approved by the INR-LGII Research and Ethics Committees (Registration No. 06/22).

Genomic DNA was extracted from peripheral blood and purified using the Puregene Blood Core Kit B (Qiagen, Hilden, Germany). The presence of the HNMT variant was assessed through restriction fragment length polymorphism (RFLP) analysis. Specifically, the C314T variant was genotyped through polymerase chain reaction (PCR) amplification of the polymorphic region within exon 4 using the following primers: 5′-GAAAAACGTTCTTTC TATCTGTTTGTATATAA-3′ and 5′-ATTTGGGCAGATCATGGTCACTTGT-3′ [25]. The PCR conditions consisted of initial denaturation at 94 °C for 5 min; 40 cycles of 94 °C for 25 s, 52 °C for 1 min, and 72 °C for 1 min; and a final extension at 72 °C for 5 min.

The resulting amplicon (394 bp) was incubated overnight with the EcoRV restriction enzyme, which cleaves the T allele into two fragments of 179 and 215 bp, while the wild-type allele is left intact. In accordance with the nomenclature of the rs11558538 variant at the cDNA or gDNA level, the allelic variants of C314T in the HNMT gene were designated as C or T. The corresponding genotypes were classified as CC, CT, or TT. Restriction enzyme digestion products were visualized on a 1.5% agarose gel, and a 1 kb molecular weight marker was included in each gel to confirm fragment size. All the samples were analyzed in triplicate. During this process, ambiguous bands were observed.

Statistical analyses were conducted using SPSS software version 23 for Windows (SPSS Inc., Chicago, IL, USA). The frequency of the HNMT gene variant was estimated by allele counting and expressed as proportions within the sample. Quantitative variables are summarized as the means ± standard deviations, whereas categorical variables were compared using Student’s *t* test for independent samples, Pearson’s χ^2^ test, or Fisher’s exact test, as appropriate. Associations between the HNMT gene variants and variables such as sex and age were evaluated using odds ratios (ORs) with corresponding 95% confidence intervals (CIs).

## 3. Results

In a sample of 18 patients, the age at diagnosis ranged from 43 to 67 years, with a mean of 58.33 ± 6.61 years, while the disease duration ranged from 4 to 25 years, with a mean of 12.50 ± 6.22 years. With respect to treatment, wide variability in therapeutic regimens was observed. Monotherapies included L-DOPA (11.1%), pramipexole (5.6%), and rasagiline (5.6%). Dual therapies included L-DOPA + pramipexole (22.2%), L-DOPA + amantadine (5.6%), pramipexole + rasagiline (5.6%), and pramipexole + amantadine (5.6%). Triple therapies included L-DOPA + pramipexole + rasagiline (11.1%), L-DOPA + pramipexole + amantadine (11.1%), L-DOPA + pramipexole + selegiline (5.6%), L-DOPA + pramipexole + biperiden (5.6%), and L-DOPA + pramipexole + rasagiline + amantadine (5.6%).

The demographic and clinical characteristics of the study population, along with group sizes, are summarized in Table 1. Significant differences were found for age and hypertension (*p* < 0.05). No significant differences were found for sex or alcohol consumption (*p* > 0.05). Although significant differences were detected for smoking (*p* = 0.011) and diabetes mellitus (*p* = 0.026), these variables could not be adequately analyzed because of the absence of cases in the control group.

To confirm the PD diagnosis in patients and evaluate controls, motor symptoms were assessed on the basis of the four cardinal features of the disease: bradykinesia, rigidity, tremor, and postural instability. All four motor variables differed significantly between the groups (Table 2). PD patients had a mean Hoehn and Yahr stage of 2.0 ± 0.51.

**Figure 1:**
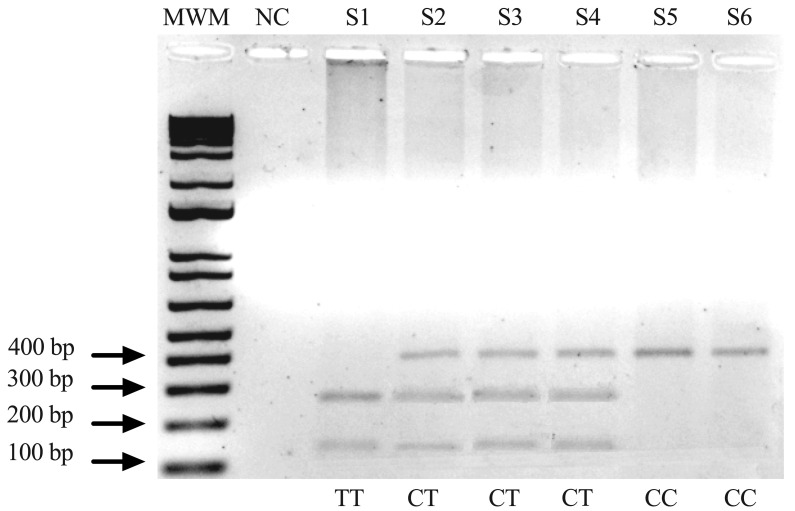
Agarose gel electrophoresis (1.5%) of RFLPs. Lane 1: molecular weight marker (MWM); Lane 2: negative control (NC); Lanes 3–8: patient samples (S1–S6). Genotypes: T/T = homozygote with restriction site; CT = heterozygote; CC = homozygote without restriction site. Abbreviations: S, patient sample; bp, base pairs.

The results of agarose gel electrophoresis for the fragments generated by EcoRV digestion of the PCR product are shown in Figure 1. The genotypic frequencies of the HNMT variants are listed in Table 3. The most frequent genotype was CC in both cases (94.4%) and controls (75.0%). The heterozygous CT genotype was observed in 5.6% of the cases and 15.0% of the controls, whereas the homozygous TT genotype was absent in the cases and was present in 10.0% of the controls. The genotype distribution was consistent with the Hardy–Weinberg equilibrium, with an exact *p* = 0.0686, although a trend toward a heterozygote deficit was observed.

Bivariate analysis did not reveal statistically significant associations between genotypes and PD in the overall sample. Nonetheless, a clinically relevant association was observed for the homozygous CC genotype, with an OR of 5.667 (95% CI: 0.593–54.115). Analysis of the allele distribution revealed that the wild-type allele (C) was associated with an increased risk of PD (OR = 7.424, 95% CI: 0.866–63.646), whereas the minor allele (T) appeared to have a protective effect (OR = 0.135, 95% CI: 0.016–1.155). When odds ratios were calculated under dominant, recessive, and additive genetic models, the observed trend suggested a protective effect of the T allele in the dominant model, although this was not statistically significant. In the recessive model, the analysis was inconclusive because the TT genotype was absent among the cases. Finally, in the additive model, a trend toward statistical significance was observed, with the T allele consistently showing a protective effect (Table 4).

In the Mantel–Haenszel stratified analysis by age, two age subgroups were evaluated. In the 51–63-year age group, the OR could not be estimated because the T allele was absent among the cases. In the 65–77-year age group, the estimated OR was 12.0 (95% CI: 0.8–160.4) (Table 5).

These findings suggest an age-related difference in allele distribution. Among individuals younger than 64 years, the T allele was absent in cases but present in controls. In contrast, among individuals older than 64 years, the T allele was present in 3.8% of the cases and 33.3% of the controls. Conversely, the frequency of the C allele in controls appeared to decrease with increasing age.

## 4. Discussion

PD represents the most common form of parkinsonism, accounting for approximately 80–90% of cases [26,27]. This subtype is typically regarded as a late-onset, largely nonhereditary disorder characterized by the classic motor features of parkinsonism [28]. The etiology of idiopathic PD remains unknown, but it is thought to result from a complex interplay of genetic and environmental factors, which likely explain most cases [29,30,31].

The high proportion of asymptomatic patients during the early stages of the disease and the lack of reliable tools to monitor or predict disease progression make early diagnosis challenging. Because PD is usually diagnosed only after the onset of motor symptoms, at which point more than 70% of dopaminergic neurons in the substantia nigra have already degenerated [32,33], our study focused on identifying a potential biomarker to enable earlier diagnosis or predict disease course.

Normal aging is the strongest risk factor associated with PD. Both the bivariate analysis and the Mantel–Haenszel stratified analysis confirmed that advanced age is a significant risk factor [34,35]. Sex has also been proposed as a risk factor, with a reported male-to-female incidence ratio of approximately 2:1. In our sample, however, females predominated, and the proportions of men and women were comparable across groups, suggesting that sex was not a determinant of disease risk [36,37]. With respect to lifestyle habits, smoking and alcohol consumption were not associated with PD. Among the systemic conditions common in the Mexican population, only hypertension was significantly associated with PD (*p* = 0.020; OR = 5.667), which is consistent with the findings of previous reports [38,39].

Genetic factors associated with PD susceptibility are increasingly being recognized. The HNMT gene, which encodes a key enzyme in histamine metabolism, has been proposed as a relevant candidate gene. Functional variants affecting histamine metabolism may influence disease pathogenesis [40,41]. While some studies have reported no association between HNMT variants and PD, others have identified links with specific haplotypes, resulting in controversial evidence [42,43].

Our findings support clinically meaningful differences in wild-type genotypes between patients with PD and healthy controls, which is consistent with the findings of previous studies [22,44]. We observed a higher frequency of the CC genotype among PD patients, which is associated with increased enzymatic activity and was consistent with previous findings [25,45]. Conversely, the less frequent CT and TT genotypes may reduce histamine metabolism in the CNS. In particular, the C allele was associated with an increased risk of PD (OR = 7.424), whereas the T allele was associated with a protective effect (OR = 0.134), which was confirmed under dominant, recessive, and additive models [46]. These results are consistent with those in previous studies regarding elevated histamine metabolite concentrations in patients with PD [25,45]. Histamine homeostasis is tightly regulated by its synthetic enzyme (histidine decarboxylase) and its metabolic enzymes (HNMT and DAO) [47]. It is therefore plausible that homozygous carriers of the wild-type allele exhibit elevated histamine metabolism, which may be counterbalanced by increased histamine synthesis secondary to the increased density of histaminergic fibers observed in PD brains, as described by Anichtchik et al. [15].

It is also worth considering the possible significance of the CC genotype (wild type) of the HNMT gene in related neurological disorders. Because HNMT plays a key role in the degradation of histamine in the brain, the presence of the CC genotype could influence the availability of histamine and consequently affect the basal ganglia circuits involved in motor control [47,48]. Although the clinical implications remain unclear, this possibility suggests that histaminergic mechanisms may contribute to the pathophysiology of parkinsonian disorders, particularly those lacking efficient therapeutic options [21].

### Limitations

The present study has several limitations. The relatively small sample size reflects the fact that the INR-LGII is not a referral center for neurological disorders; many patients were treated for unrelated conditions such as fractures, hearing loss, or cardiac problems, which limited recruitment. In addition, the small sample size prevented the stratification of analyses according to PD subtypes because this could lead to empty cells in the contingency tables required for odds ratio (OR) calculations. Another limitation is that ancestry markers were not analyzed, which should be taken into account when interpreting the genetic findings. In addition, the lack of analysis of nonmotor symptoms—particularly constipation, hyposmia, and depression—represents a limitation, even though sleep disturbances are the nonmotor feature most strongly linked to PD. Finally, blood pressure measurements could not be confirmed, despite hypertension being statistically significant in our analysis. However, one strength of this study is the use of clinical and neurological criteria for classifying patients with PD.

## 5. Conclusions

Our findings suggest that individuals carrying the C-allele genotype (wild type) of the HNMT gene may have an increased risk of developing Parkinson’s disease, whereas the T-allele may confer a protective effect. In addition, advanced age and hypertension were identified as potential risk factors for the disease. Given that PD is a multifactorial disorder and considering the limited sample size of the present study, these findings should be regarded as exploratory and require confirmation in larger independent cohorts.

## Figures and Tables

**Table 1:** Demographic and clinical characteristics of the study population.

	PD (*n* = 18)	Controls (*n* = 20)	OR (95% CI)	Statistic	*p*-Value
Age mean ± SD	66.67 ± 5.204	59.70 ± 6.062	1.84 (3.23–10.70)	*t* = 3.780	0.001
Age group: 51–63 years *n* (%)	5 (27.8)	14 (70.0)	0.16 (0.04–0.67)	χ^2^ = 6.75	0.008
Sex (f/m)	11 (61.1)/7 (38.9)	13 (65.0)/7 (35.0)	1.18 (0.32–4.42)	χ^2^ = 0.062	0.804
Smoking *n* (%)	5 (27.8)	0 (0.0)	Not estimable	χ^2^ = 6.397	0.011
alcohol intake *n* (%)	11 (61.1)	11 (55.0)	1.29 (0.353–4.689)	χ^2^ = 0.145	0.703
Hypertension *n* (%)	9 (50.0)	3 (15.0)	5.667 (1.22–26.33)	χ^2^ = 5.371	0.020
Diabetes Mellitus *n* (%)	4 (22.2)	0 (0.0)	Not estimable	χ^2^ = 4.967	0.026

Abbreviations: PD, Parkinson’s disease; OR, odds ratio; CI, confidence interval; SD, standard deviation; f/m, female/male; χ^2^, chi-square.

**Table 2:** Clinical Symptoms of Parkinson’s Disease.

Symptom	PD (*n* = 18)	Controls (*n* = 20)	OR (95% CI)	Statistic	*p*-Value
Bradykinesia *n* (%)	18 (100.0)	0 (0.0)	Not estimable	χ^2^ = 38.000	0.000
Rigidity *n* (%)	18 (100.0)	1 (5.0)	19.00 (2.82–128.01)	χ^2^ = 34.200	0.000
Tremor *n* (%)	14 (77.8)	1 (5.0)	66.50 (6.68–661.61)	χ^2^ = 21.002	0.000
Postural instability *n* (%)	10 (55.6)	0 (0.0)	Not estimable	χ^2^ = 15.079	0.000
Hoehn & Yahr scale	2.0 ± 0.51	--	--	--	--

Abbreviations: PD, Parkinson’s disease; OR, odds ratio; CI, confidence interval; χ^2^, chi-square; Hoehn & Yahr, clinical disability scale.

**Table 3:** Risk of Developing Parkinson’s Disease in the Presence of the Polymorphism.

Genotype	PD (*n* = 18)	Controls (*n* = 20)	OR (95% CI)	Statistic	*p*-Value
CC *n* (%)	17 (94.4)	15 (75.0)	5.667 (0.59–54.11)	χ^2^ = 2.694	0.101
CT *n* (%)	1 (5.6)	3 (15.0)	0.333 (0.03–3.53)	χ^2^ = 0.897	0.344
TT *n* (%)	0 (0.0)	2 (10.0)	Not estimable	χ^2^ = 1.900	0.168
Alleles					
C *n* (%)	35 (97.3)	33 (82.5)	7.424 (0.866–63.646)	χ^2^ = 4.360	0.037
T *n* (%)	1 (2.7)	7 (17.5)	0.134 (0.016–1.155)		

Abbreviations: PD, Parkinson’s disease; OR, odds ratio; CI, confidence interval; χ^2^, chi-square; C, cytosine; T, thymine.

**Table 4:** Association of the HNMT C314T polymorphism with Parkinson’s disease.

Model/Comparison	PD (*n* = 18)	Controls (*n* = 20)	OR (95% CI)	*p*-Value
Dominant (CT + TT vs. CC) *n* (%)	CT + TT: 1 (5.6)/CC: 17 (94.4)	CT + TT: 5 (25.0)/CC:15 (75.0)	0.17 (0.018–1.685)	0.184
Recessive (TT vs. CC + CT) *n* (%)	TT: 0(0.0)/CC + CT: 18 (100)	TT: 2(10.0)/CC + CT: 18 (90.0)	Not estimable	0.488
Additive (allelic) (T vs. C) *n* (%)	T: 1 (2.8)/C: 35 (97.2)	T: 7 (17.5)/C:33 (82.5)	0.135 (0.016–1.155)	0.059

Notes: Percentages in the allelic model were calculated based on the total number of alleles (2N): cases = 36 alleles; controls = 40 alleles. *p*-values were calculated using a two-tailed Fisher’s exact test. Abbreviations: PD, Parkinson’s disease; OR, odds ratio; CI, confidence interval; C, cytosine; T, thymine.

**Table 5:** Mantel–Haenszel stratified analysis by age and HNMT C314T alleles.

Age Group	Allele	PD (*n* = 18)	Controls (*n* = 20)	OR (95% CI)	*p*-Value
51–63 years *n* (%)	C	5 (100)	12 (85.7)	0.70 (0.5–0.9)	0.25
	T	0 (0.0)	2 (14.3)		
65–77 years *n* (%)	C	12 (92.3)	3 (50.0)	12.0 (0.8–160.4)	0.04
	T	1 (7.7)	3 (50.0)		

Notes: Mantel–Haenszel test = 2.56 (*p* = 0.09); Cochran test = 4.47 (*p* = 0.02). Abbreviations: PD, Parkinson’s disease; OR, odds ratio; CI, confidence interval; C, cytosine; T, thymine.

## Data Availability

The raw data supporting the conclusions of this article will be made available by the authors on request.
